# Sprinting into the field of neuro-ophthalmology from the streets of Brooklyn

**DOI:** 10.1186/s40673-020-00118-w

**Published:** 2020-07-20

**Authors:** Steven L. Galetta

**Affiliations:** Departments of Neurology and Ophthalmology, New York University Grossman School of Medicine, 222 East 41st Street, 14th Floor, New York, NY 10017 USA

## Growing up

**Fig. 1:**
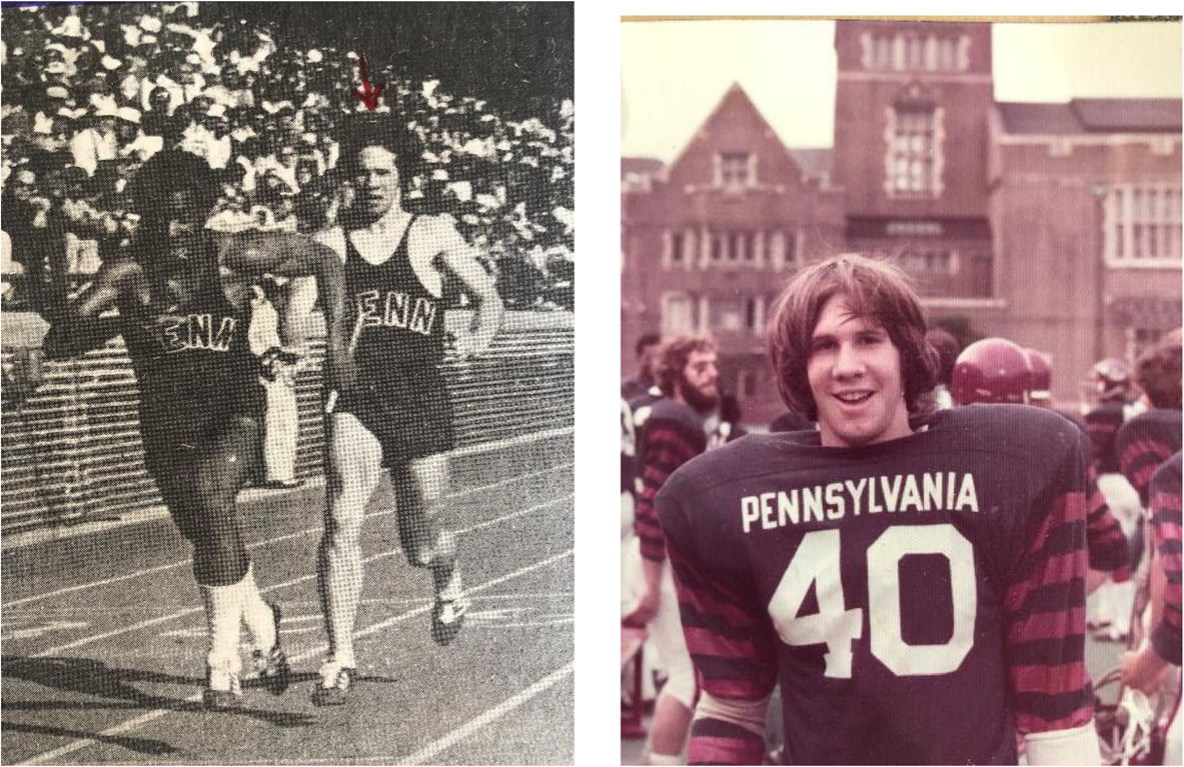
**a** running in the 4X 200 at the Penn Relays. **b** Needing a haircut before a football game

I grew up in Brooklyn, NY, in the concrete jungle. Everything we did was measured by sewers, those manhole covers that periodically occur on each city block. Our football field, running track, stickball field and slap ball venue were all measured or created by using the sewers as a landmark. For instance, how far you could hit a baseball was measured in sewers. Running was an important element of everything you did, both for playing sports and in avoiding predators. I was blessed with speed, and that carried me to Xaverian High School in Brooklyn and then to the University of Pennsylvania (Penn). I was fortunate to be on a 4X 100 relay that finished 8th in the country in 1977 and our unit was consistently one of the best teams on the East Coast of the United States (Fig. 1a). At Penn, I also played sprint football, a varsity sport that requires an athlete to be under a certain weight several days prior to the game. I played running back and captained the 1978 team (Fig. 1b). Playing sports taught me many valuable lessons about perseverance, overcoming adversity and teamwork. Sports taught me how to get the best out of myself and, perhaps more importantly, out of the people on my team. To be successful, you really have to maximize and appreciate your teammates strengths. Not everyone can do what you want them to do. Repetitively focusing on their weaknesses is usually a losing game.

## Medical school

In 1979, I entered the Cornell University Medical School. I remember looking to the right and left of me on the first day of school and concluding that I was probably the dumbest person in my class. I worked hard and nothing really came naturally to me. There was one exception; that was neuro-anatomy. For some reason, I was able to visualize the pathways and the nuclei well. This was the case even though I was lousy at puzzles. For the first time, people would ask me questions rather than me asking them. Like most things in life, you try to do more of what you are good at and limit those areas for which you have less talent. You only have to be good in one area to be successful, but you have to maximize your potential by working hard. Very few people can be consistently good at something without a lot of effort. During the very last rotation in medical school, I worked in ophthalmology and thought it was great. I said to myself at the time, “if I had been exposed to this area sooner, I might have been an ophthalmologist.” Nonetheless, I had already matched in neurology.

## Neurology residency

In 1983, I started internship in medicine followed by neurology residency at the University of Pennsylvania. A couple of fortuitous things happened to me in my first year as a neurology resident. First, the Wills Eye Group in neuro-ophthalmology admitted all their inpatients to Penn. This service was led by Drs. Norman Schatz and Peter Savino. The patient volume was so high that there was a whole service dedicated to neuro-ophthalmology. I did everything I could to be on that service, and would switch rotations with one of my colleagues who loved neuromuscular disorders. One of the highlights of each week was neuro-ophthalmology rounds with Dr. Norman Schatz. These rounds occurred on Tuesday mornings, and Dr. Schatz would insist on bringing patients to a conference. He would examine the patient and discuss their problem and, then, draw the relevant anatomy on the chalk board. The rounds were memorable because Dr. Schatz is one of the finest clinicians I have ever met. He taught me instant neurological diagnosis. Dr. Schatz could make the diagnosis of a complicated problem in seconds. His personality is also larger than life, and he is so enjoyable to be around. Our chairman of neurology at Penn at the time, Dr. Donald Silberberg, was also a neuro-ophthalmologist and a multiple sclerosis (MS) expert. So, the training experience was quite concentrated, with a diversity of neuro-ophthalmology mentors. I think the important point here is that you need to engage with your mentors. The mentee must play an active role and seek opportunities.

The second important encounter with a great mentor at the end of my first year as a neurology resident was with Dr. Robert I. Grossman. He was the chief of neuro-radiology at Penn at that time. Dr. Grossman was an early leader in sequence development for MRI and, in my opinion, he is a true genius. I asked Dr. Grossman at the end of my first year of neurology if there was project that I could work on. He said “sure, we have this compound called gadolinium and we need to study it in humans.” So, patients with MS were chosen, and this was nearly a decade before any immunomodulatory therapy was available. My job was to admit the patients to a clinical research unit and to examine them carefully. No one suffered any side effects, and there was remarkable correlation of new demyelinating lesions on MRI with patients’ examination findings. The attending neurologists and radiologist would joke that I did many of the physical tasks of the study, including parking the patients’ cars! Being a sprinter came in handy yet again. These were formative experiences and explain my subsequent and current interests in neuro-ophthalmology and MS. The relationship with Dr. Grossman was particularly important. We became close friends, and he would become invaluable mentor throughout my life. In those days, mentorship was not highly formalized, but you gravitated to those people that made you better as person and as an investigator and clinician. I would bring normal brain MRI films to Dr. Grossman just to get his opinion. He helped so many patients that he never met. More about him later… If you are going to succeed in medicine, you have to say “yes” far more than you say “no,” particularly if certain people ask you for assistance. The mantra of “just say no” early in your career to focus on your work is usually not correct. It is the relationships you build that are most important.

## Fellowship

**Fig. 2:**
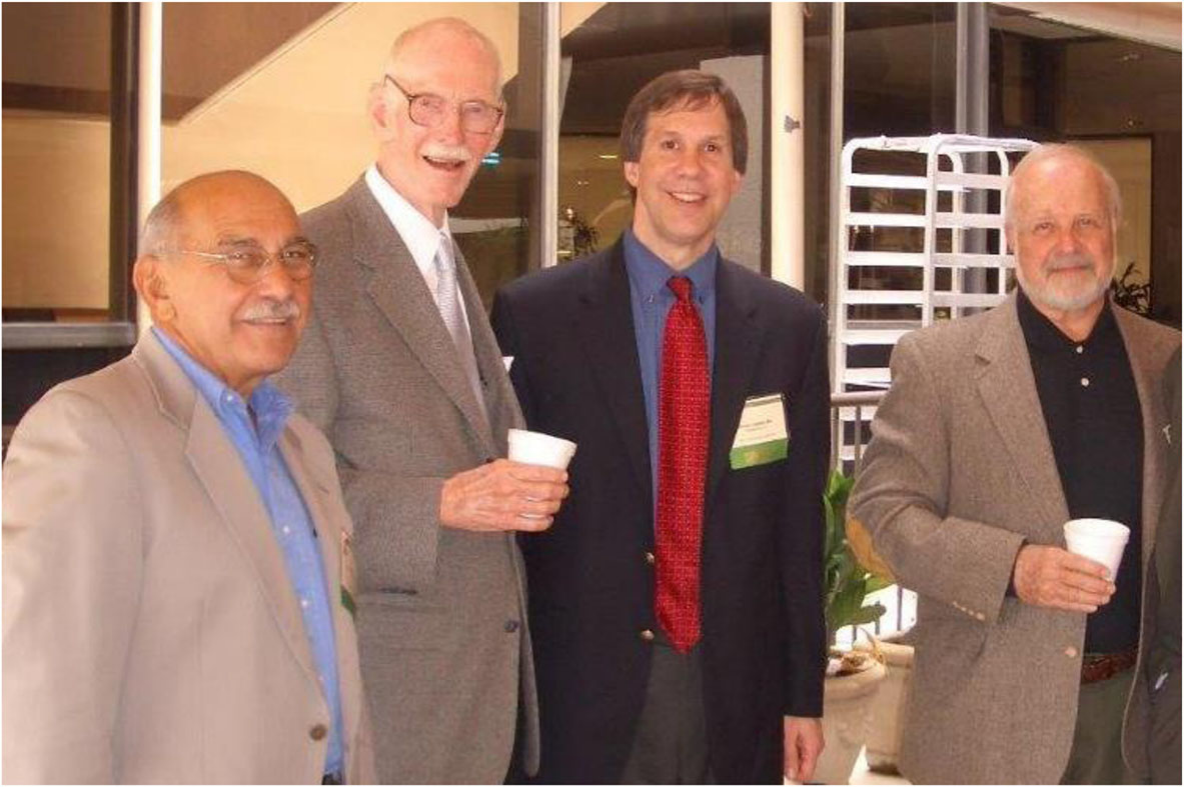
My neuro-ophthalmology mentors from the left- Drs. Norman Schatz, Lawton Smith and Joel Glaser

I did my fellowship at the Bascom Palmer Eye Institute. Remarkably, Dr. Norman Schatz had now moved to Miami to join Dr. Joel Glaser and Dr. J. Lawton Smith as my neuro-ophthalmology mentors (Fig. 2). It was a great experience, because Dr. Schatz was an unbelievable clinician and Dr. Glaser was a scholar and great writer. Dr. Smith was larger than life, and he taught me how to examine patients in a multitude of ways. It was during this time that I forged another important relationship; this was with Dr. Nancy Newman at Emory University. We had both just finished our fellowships, and we vowed to help each other. For the next several decades, we worked to give annual courses at the American Academy of Ophthalmology and American Academy of Neurology. This emphasizes the fact that you should affiliate yourself with people that are smarter or better than you are. Dr. Newman is one of those individuals.

## Penn faculty

**Fig. 3:**
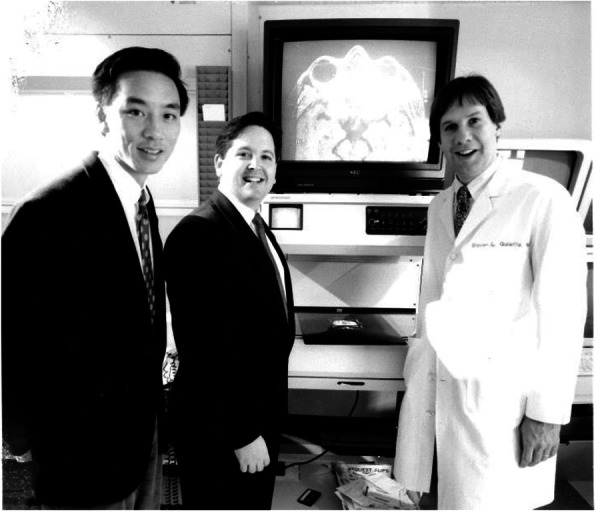
From left, Drs Grant Liu and Nick Volpe early in our careers

**Fig. 4:**
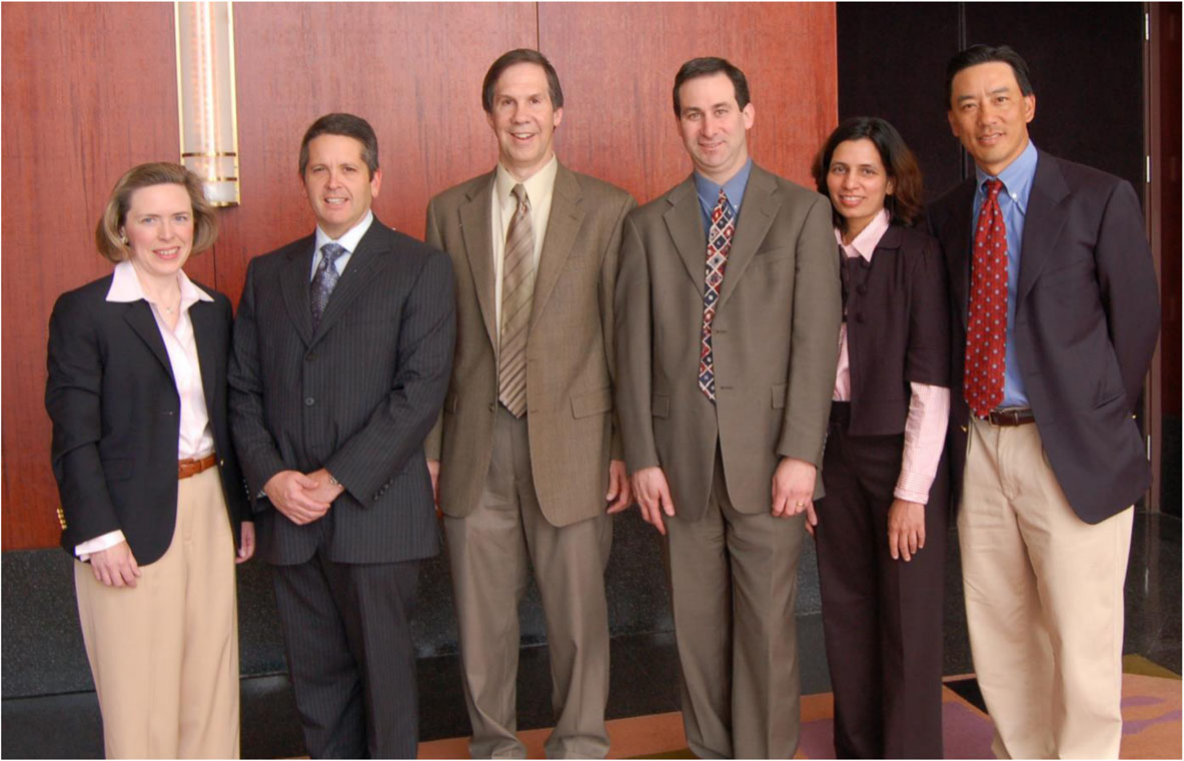
The Penn team in the early part of this decade. From left, Drs Laura Balcer, Nick Volpe, me, Ken Shindler, Madhura Tamhankar and Grant Liu

After fellowship, I returned to Penn to become the director of neuro-ophthalmology. My neuro-ophthalmology practice grew quickly and I really enjoyed teaching. In 1989, I became the neurology residency program director. No one really wanted the job at that time because you did not get paid for it and you did the work in your spare time. However, the best jobs can be the ones that no one wants. I honed my teaching skills during this period as I felt personally responsible for the fund of knowledge that our neurology residents were offered and mastered. Teaching and learning also require teamwork. In education’s most perfect form, the distinction between who is teaching and who is learning is completely blurred. I have learned far more from my colleagues and trainees, and it has been an honor to be their teammate. At the same time, my neuro-ophthalmology practice continued to grow rapidly. Fortunately, in 1993, Drs. Nicholas Volpe and Grant Liu joined me in the neuro-ophthalmology practice (Fig. 3). It was the perfect combination. Dr. Volpe was an ophthalmologist and Dr. Liu had an interest in pediatric ophthalmology. So, we each had our strengths. However, we vowed to support each other and, by then, we were ready to develop a fellowship program. One of our first fellows in 1996 was Dr. Laura Balcer, who became a neuro-ophthalmologist and an epidemiologist. She joined our neuro-ophthalmology team as the fourth member. Dr. Balcer was an invaluable member of our team and brought scientific rigor. It was during this time that clinical trials were very active in MS and clinically isolated neurological presentations such as optic neuritis. We realized that vision had not been incorporated into the assessment of patients with MS despite the frequent occurrence of visual symptoms. Dr. Balcer set out to determine what might be the best test to distinguish patients with MS from disease-free controls. Low-contrast letter acuity emerged as that best single visual test and it shortly became incorporated into pivotal MS trials, including the initial clinical trials of natalizumab [1–6]. Unlike high-contrast letter acuity (visual acuity testing in black and white), which did not show the ability to detect treatment effects, low-contrast letter acuity (acuity testing in shades of gray) could distinguish treatment groups in the direction of favoring the active agent [1, 2]. This study also demonstrated that there was a fair amount of hidden visual disability in MS, emphasizing that your investigation is only as good as the measurement systems you employ. In the 2004 to 2005 timeframe, optical coherence tomography (OCT) had emerged as a new technology and we employed it in the MS population. We found that eyes showed retinal nerve fiber layer thinning in patients with MS compared to controls, even in the absence of acute optic neuritis. This suggested that there was silent disease activity that was not being measured. Likewise, we found that there was visual loss and retinal nerve fiber layer loss in patients with so called “benign MS” and in patients with MS followed over time [4]. This demonstrated once again that there was hidden visual disability in MS. We began working with Dr. Joel Schuman, one of the co-inventors of OCT technologies for the eye. We now worked with his group to segment the retinal layers and measure their thickness in patients with MS. Our first published study in this area showed that retinal ganglion cell layer thickness in the macula correlated best with low-contrast letter acuity and with vision-related quality of life measures [7]. Meanwhile, Dr. Grant Liu spearheaded an effort to write a textbook, entitled *Neuro-Ophthalmology: Diagnosis and Management*. Co-authors were Dr. Nicholas Volpe and myself. Dr. Liu was the reason that we were successful in producing this work, again emphasizing that you should work in teams. Drs. Ken Shindler and Madhura Tamhankar joined us to make 6 neuro-ophthalmologists and all of us prospered as team (Fig. 4). We then became interested in the area of concussion, a condition that was gaining increased attention because of the potential short- and long-term consequences. We applied the principles we had learned about testing in MS to the field of concussion. This is an important point. One should look at what disease is emerging in your field and apply what you know from other conditions to that disorder. Like MS, there really was not much attention being paid to the visual changes associated with concussion. Dr. Laura Balcer and I decided that we would examine visual testing in concussion. In 2010, we began looking at rapid number naming testing; this evolved to rapid picture testing and other aspects of the underpinnings of ocular motility disorders in concussion [8–11].

## NYU faculty

When Dr. Grossman left Penn in 2000 to become chair of radiology at the NYU School of Medicine, it was a big loss for all of us. He was a true star. It did not take long before Dr. Grossman was named the Dean and the CEO of the NYU School of Medicine and Medical Center in 2007. His strong relationship with the chairman of the Board of Trustees, Ken Langone (founder of Home Depot), brought great prosperity to the medical center as the institution dramatically climbed higher in every aspect of medicine. The medical center was subsequently named after Mr. Langone and the medical school was recently named after Dr. Grossman. Shortly after his appointment as Dean and CEO in 2007, Dr. Grossman began to recruit me to NYU. However, I loved my jobs at Penn, which included being the residency program director for over 20 years and the fellowship director for neuro-ophthalmology. I also was now the Associate Dean of Admissions for University of Pennsylvania School of Medicine and the Vice Chair of Neurology. I was doing what I could do best, which was to build teams and recruit talented people. By that time, we had recruited some of the most talented neurology residents at Penn into neuro-ophthalmology. Fifteen Penn residents went on to become neuro-ophthalmologists, and many of them are leaders across the country. During this time, I developed some rules that governed how I thought about clinical care, education and leadership (Table 1, 2 and 3).

**Table 1:** The Most Important Clnical Pearls Learned Along the Way

**Second Opinions**	**• A good thing** **• Sharing information is the key to learning and teaching** **• Not a sign of weakness, but a sign of confidence!** **• Require and demonstrate teamwork**
**Tough Medical Decisions**	**• Even with scientific proof, the treatment options for your patient may not be clear** **• You must always decide how you would want to be treated…** **• …or how you would treat a family member, friend or colleague** **• Listen carefully to the patient as they will often lead you out of the dilemma!**
**The Real Problem is Usually Greater than the Potential One**	**• Don’t get overwhelmed by the consequences of medical therapy and leave the primary problem untreated** **• It is your job to help the patient understand the risks of the disease and the treatment** **• Be honest at all times** **• It is okay to say “I don’t know”**
**Watch How Your Colleagues Act**	**• It is okay to incorporate what they do, how they say and how they write it** **• Copying effective behavior can lead to good care** **• You will be judged by communication skills** **• Remember the A’s of the effective colleague and physician – Affability, Availability, Attentiveness, Ability, Acumen**
**Healthy Relationships**	**• Affiliate yourself with people that make you better as a doctor and person** **• Avoid people who make you feel inferior** **• Hang out with people who are smarter than you – it is the only way to get better** **• One of the greatest rewards is to help a colleague improve their knowledge**
**Nonorganic or Organic?**	**• There is almost always an emotional component to any illness** **• Your job is to try to figure out those percentages – it is easy when the emotional component is small** **• When it is 50–50, be very careful!** **• Don’t get frustrated and be compassionate** **• There is almost always a way to get your point across while being reassuring**
**Be Happy for Your Colleagues!**	**• The most unhappy doctors need their competitors to fail to feel successful** **• Don’t waste your neurons on unproductive thoughts** **• Don’t worry – medicine will humble each of us on more than one occasion!**
**Learn More**	**• No matter how much you know, be prepared to learn more** **• The effective clinician is a student for life** **• There can be errors of omission and commission – be open!**
**Try to Read Your Patient**	**• Watch how they react to what you are saying, make the necessary adjustments** **• Fight hard for your patients, let them know you are battling for them** **• Be a passionate observer!**
**Your Personal Life is Important**	**• Work hard to show your family and friends how important they are to you** **• Try to do your best, no matter what that looks like!**

**Table 2:** Top Ten Education Tips

**Be brief – the best 30-min talk is given in 15 min**	
**You could ruin a good talk by going over the allotted time**	
**No more than 30 words per slide**	
**Not what you say, it’s how you say it**	
**Be dumb, get to a basic level and build from there**	
**Give a road map, tell the audience where you are headed**	
**Avoid Um’s and Uh’s – take a breath instead!**	
**Talk to a few people in the room, head-nodders are always helpful to look at**	
**Get audience to participate**	
**Don’t read your slides**

**Table 3:** Being a Leader - Why Do It?

**For the chance to build something bigger and better**	
**To help junior faculty succeed in their careers**	
**To build highly-functioning and integrated teams to serve a multidisciplinary function**	
**To figure out the future direction of the field and to be a part of those changes**	
**To improve training for the medical students and residents – neurologists of the future!**	
**Being a chair has taught me how fragile things can be, and how one individual can either make or break a team**	
**Don’t hesitate to properly engage those individuals that drag the team down!**	
**Everything is a compromise and it may take multiple attempts to get what you need**	
**Some things are worth fighting for – for those less important, you just have to let go**	
**It is about everyone else and much less about you – don’t do it if is about you – you will fail**	

After a 4 year courtship, I gave in to Dr. Grossman’s request to join him as the chair of neurology at the NYU Grossman School of Medicine in 2012. I never thought I would leave my job at Penn, but Dr. Grossman said something to me that resonated strongly. “You could stay in your current position and that would be fine, but if you really wanted to challenge yourself, you would come here and help build neurology,” he said. Sometimes you can be afraid to leave your comfort zone, but your greatest learning experience can be to do exactly that. In 2012, Dr. Laura Balcer joined me as the Vice Chair of Neurology and off to NY we went. We have been part of two different major disasters: Superstorm Sandy and the COVID-19 pandemic. Both of these events were extremely challenging, but for different reasons. Hurricaine Sandy wiped out our hospital system because of extraordinary flooding, and COVID-19 rocked NYC because of the quick and extensive spread of infection.

## Superstorm Sandy and NYU neurology development

Superstorm Sandy arrived to NYC on October 29, 2012, and produced massive flooding. I had arrived to NYC the following day to move into my new apartment. I was living in the East Village in an NYU-owned apartment on the 9th floor. First, there were no working traffic lights and it was like a bumper cars ride through the city. Cars would enter the middle of the street and it was not clear who should go first. The angled streets of the East Village did not make the task any easier. Once arriving at my apartment, I realized that there were no elevators and there was no electricity. Climbing up and down 9 flights of steps was a near death experience. That storm was physically destructive, but we did not lose any lives at NYU Langone. This was a tremendous miracle. Hardship did abound but we were able resume outpatient services in a matter of days at more than the 90 ambulatory sites in the NYU system at that time. It was the hospitals that suffered this time, and, once again, innovation led the way out of the siege. There were millions and millions of gallons of water in our three hospitals - Tisch, Bellevue and the Veteran’s Administration (VA) Hospital - that needed to be pumped out. NYU was, unfortunately, at the bottom of a saucer filled by a fourteen foot storm surge. As I approached the medical center on my first day on the job as chair of neurology on November 1, 2012, I could only wonder what I had gotten myself into. The very first chairs’ meeting was held in its typical location, but there was no electricity and the room smelled badly of oil. The oil vaults in the basement had busted. In an odd twist, the most important thing in this disaster had been to get the patients out of our hospitals and transported safely to other medical centers. Many of the most critical patients during the Sandy crisis had to carried out in the stairwells by the creation of a human chain.

**Fig. 5:**
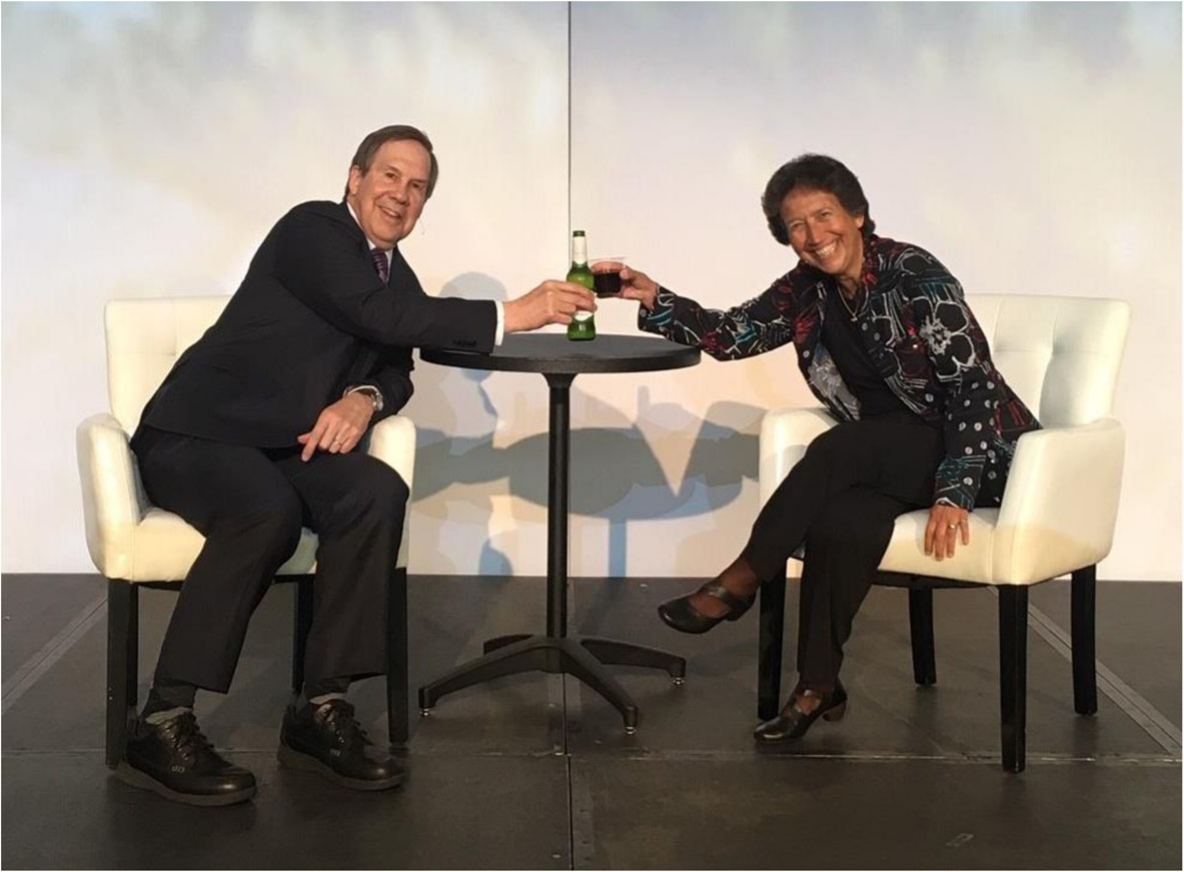
Toasting my colleague Dr. Nancy Newman who was being interviewed as one of the giants in neurology

**Fig. 6:**
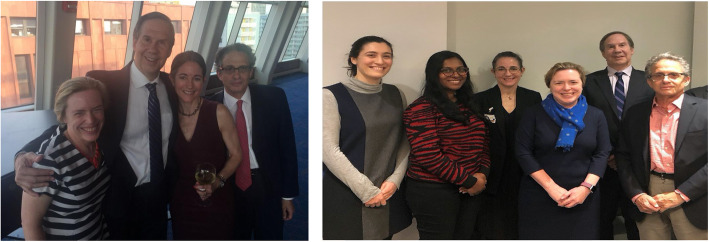
left showing the NYU team- from left, Drs Laura Balcer, me, Janet Rucker and Floyd Warren. B- team expands to include Drs. Doria Gold and Cinthi Pillai

After the storm receded, many of the physicians were redeployed and we had to quickly find spots for our residents to train. We were able to redeploy them at Harlem Hospital and at the Brooklyn VA. A group also went into the outpatient practices. This was a black swan event just like the coronavirus pandemic of today. For the most part, these events bring out the best in people, and they demonstrate the tremendous resilience and spirit of the attending physicians, residents and staff. Very few complained of hardship and almost everyone pitched in. Like the COVID-19 pandemic, there was opportunity to rebuild from scratch. Everyone was brought together after the storm in what was then a small department, and we could begin to operate as a whole. The medical center also had its challenges to reopen after Sandy. The two big obstacles besides the water in the basement were diagnostic imaging and food. There were no MRI machines as they had been destroyed in the basement by water. Portable MRI machines were then delivered by cranes to a courtyard area so we could image our inpatients. We also needed a cafeteria and a galley to prepare patients’ meals. A portable kitchen area was created on the roof of one our buildings. These efforts enabled the Tisch Hospital to reopen 6 weeks later. Because of the solid financial shape of NYU Langone, all of the employees were paid during this 6 week period of no inpatient care. One of the most important things I learned about this disaster was that one should also look for opportunity in times of adversity. For the next 8 years, the buildout of NYU neurology was relatively smooth. We grew enormously and added over 180 neurologists to the department and developed 9 new divisions including neuro-intensive care and, of course, neuro-ophthalmology. The residency program grew from 7 to 11 residents per year, and we developed a second neurology residency training program in Brooklyn that has now grown to five residents per year. We built neuro-ophthalmology at NYU and Dr. Janet Rucker joined us to lead the division.  Dr. Rucker was trained by Dr. Nancy Newman (Fig. 5). Shortly thereafter, Dr. Floyd Warren joined our group as did Drs. Cinthi Pillai, Doria Gold and Scott Grossman. The neuro-ophthalmology fellowship prospered and four NYU residents in addition to outside trainees joined us. Laura Balcer then went on to become the editor in chief of the journal neuro-ophthalmology, a position she holds today (Fig. 6).

## COVID-19 and New York

On March 1, 2020, the first confirmed case of COVID-19 was announced. A health care worker who had visited Iran was now quarantined in her Manhattan home. On Monday March 2, 2020, NYU Langone Health and the NYU Grossman School of Medicine took the unprecedented action of banning out of state and out of country travel to meetings. I was scheduled to be the Jacobson Lecturer at the upcoming North American Neuro-Ophthalmology Society (NANOS) annual meeting that started on March 6, 2020, in Amelia Island, Florida. A great deal of controversy ensued during the next several days; many NANOS members thought that the NYU travel edict was an overreaction. People did not seem to understand that the Centers for Disease Control (CDC) had already fallen behind in this evolving pandemic, and that this ban was the right thing to do. Many colleagues were looking for guidance from the CDC, but the CDC itself was scrambling to find its footing regarding what to do. They had lost the opportunity to do early surveillance for the COVID virus because of the availability of testing. In the ensuing days, all the NY medical institutions enacted travel bans to meetings. The bans soon extended to other institutions throughout the country. The NANOS meeting was held, and approximately 500 attendees were able to make it. It was to be one of the last large scale medical meetings to be held. Zoom conferencing was installed for those of us who could not attend NANOS. I gave my first lecture by Zoom and apparently it went well. It was a bit strange to be talking to one’s computer without seeing the faces of your colleagues.

Over the next several days, it had become clear that the virus was rapidly spreading throughout NYC. We quickly converted to Webex and videoconferencing for virtually all of our conferences. I presented the last fully attended grand rounds on the topic of optic neuritis on March 10, 2020. I substituted for a speaker who was originally was coming from California to give a talk on behavioral neurology. It has become clear in this crisis that face-to-face meetings are not always necessary, and it is likely that many of us will attend meetings virtually going forward.

We began the rapid deployment of video visits and telephone visits in lieu of face-to-face clinical visits whenever possible. This was an auspicious start to telemedicine in NYU neurolog say the least. We had just gone from zero to 100 miles per hour with our telemedicine effort. In many ways, the COVID-19 pandemic was similar to Superstorm Sandy in that new information systems were implemented: telemedicine with COVID-19 and Epic with Sandy. In early 2013, immediately after Sandy, Epic was initiated; in 2020, telemedicine quickly emerged at our institution. The downtime associated with Sandy allowed us to learn Epic; similarly, the downtime of this pandemic has allowed us to learn the nuances of video visits. Dr. Neil Busis, who had recently joined the NYU faculty, taught us about the platforms that we could use, including Haiku on the iPhone and Canto on the iPad. We had to learn a whole new set of codes for video visits and telephone visits. Over the ensuing days, we learned more about the virus and the potential therapies. However, there were largely untested remedies that were being touted. We went to skeleton crews to limit the attending and resident physician exposures. Despite social distancing techniques, the numbers of cases continued to increase in New York and the number of patients requiring ICU beds with acute respiratory distress syndrome (ARDS) exploded. It would take just a week for virtually every bed in our hospital to be converted to a COVID-19 bed. Neurological admissions became fewer as patients stayed away and the needs for infusing patients with steroids and monoclonal antibodies declined during this period. Soon, our neurology residents were redeployed to the medical services given patient volumes in the ICU and on the medicine service. Novel ideas emerged to preserve and refurbish the much needed N95 masks. Institutions were desperate for personal protective equipment (PPE) and ventilators. Although some of these requests were largely out of our control, our supply chain was greatly augmented by Mr. Ken Langone’s connections to industry and the government. The ingenuity of the staff to convert operating room ventilators to support medically ill patients was also important. During this period, I would frequently look out my NY window at the many tall apartments in NY and wonder if we were all locked down in our own cruise ships, on which the virus could spread.

Although there were there were many differences in these two major natural disasters, the most significant similarity was the heroic response of our physicians, nurses and staff. Although fear was appropriately profound in both cases, we were impressed with how these periods of uncertainty inspired courage, hope and resilience in our colleagues. Even in times of social distancing, the value of teamwork and leadership were never more important. No one really achieves anything alone, and it would be no fun if we did.
